# Right Ventricular Function in Long-Term Survivors of Childhood Acute Lymphoblastic Leukemia: From the CTOXALL Study

**DOI:** 10.3390/cancers15215158

**Published:** 2023-10-26

**Authors:** Gloria Heredia, Rafael Gonzalez-Manzanares, Soledad Ojeda, Jose R. Molina, Consuelo Fernandez-Aviles, Francisco Hidalgo, Jose Lopez-Aguilera, Manuel Crespin, Dolores Mesa, Manuel Anguita, Juan C. Castillo, Manuel Pan

**Affiliations:** 1 Cardiology Department, Reina Sofia University Hospital, 14004 Cordoba, Spain; 2 Maimonides Institute for Research in Biomedicine of Cordoba (IMIBIC), 14004 Cordoba, Spain; 3 Hematology Department, Reina Sofia University Hospital, 14004 Cordoba, Spain

**Keywords:** cardio-oncology, cardiotoxicity, right ventricular strain, echocardiography, childhood cancer survivor

## Abstract

**Simple Summary:**

Childhood cancer survivors have an increased lifetime risk of cardiac problems due to the cardiotoxic effects of cancer treatments. While most research has focused on the left side of the heart, little is known about the effects of these treatments on the right-side chambers. We used cardiac ultrasound to examine the right ventricle in 90 long-term survivors of childhood leukemia and a control group of 58 healthy siblings. Our findings showed that a noticeable number of survivors had subtle alterations in the pump function of the right ventricle, especially when measured using a novel parameter called a free-wall strain. Alterations in free-wall strain were more common in the survivors who were obese or smoked. This study underscores the importance of incorporating right ventricular strain assessment into routine cardiac surveillance and promoting healthy lifestyle habits among survivors.

**Abstract:**

There are limited data regarding right ventricle (RV) impairment in long-term survivors of childhood acute lymphoblastic leukemia (CLS). The aim of this study was to assess RV function in these patients using echocardiographic conventional measurements and automated RV strain. Echocardiographic recordings of 90 CLS and 58 healthy siblings from the CTOXALL cohort were analyzed. For group comparisons, inverse probability weighting was used to reduce confounding. The CLS group (24.6 ± 9.7 years, 37.8% women) underwent an echocardiographic evaluation 18 (11–26) years after the diagnosis. RV systolic dysfunction was found in 16.7% of CLS individuals using RV free-wall strain (RVFWS) compared to 2.2 to 4.4% with conventional measurements. RV systolic function measurements were lower in the CLS than in the control group: TAPSE (23.3 ± 4.0 vs. 25.2 ± 3.4, *p* = 0.004) and RVFWS (24.9 ± 4.6 vs. 26.8 ± 4.7, *p* = 0.032). Modifiable cardiovascular risk factors such as obesity (*p* = 0.022) and smoking (*p* = 0.028) were independently associated with reduced RVFWS. In conclusion, RV systolic function impairment was frequent in long-term survivors of childhood leukemia, underscoring the importance of RV assessment, including RVFWS, in the cardiac surveillance of these patients.

## 1. Introduction

Acute lymphoblastic leukemia (ALL) is the most prevalent type of cancer in the pediatric population. Over the past few decades, anthracycline-based therapies have significantly improved survival rates in these patients [1]. However, due to the exposition to cardiotoxic agents, childhood leukemia survivors (CLS) have an increased risk of cardiovascular disease during their lifetimes [2].

The main mechanism involved in the pathogenesis of anthracycline-related cardiomyocyte injury is the inhibition of topoisomerase 2β, leading to mitochondrial dysfunction, increased reactive oxygen species production, and apoptosis. Other proposed mechanisms are reduced ATP generation, direct damage to the mitochondria, the lipid peroxidation of cell membranes, and changes in calcium and iron homeostasis [3,4,5].

Most echocardiographic surveillance studies have focused on left ventricle evaluation [6,7], with the right ventricle (RV) being less frequently assessed in CLS [8]. Although RV function has been shown to be strongly associated with outcomes in multiple cardiac conditions, its quantification traditionally poses challenges because of the complex geometry and physiology of the RV [9]. Recently, right ventricle strain (RVS) has been proposed as a straightforward and robust measure of right ventricular function [10]. Importantly, RVS is angle-independent and has a stronger correlation with cardiac magnetic resonance-based RV ejection fraction than conventional echocardiographic indices [11].

The aim of this study was to assess RV function in a cohort of long-term CLS through the quantification of automated RVS and conventional parameters.

## 2. Materials and Methods

### 2.1. Study Design and Participants

This ancillary study is integrated into the framework of the CTOXALL study, a cross-sectional study that recruited a cohort of long-term childhood cancer survivors who had been diagnosed with ALL between 1985 and 2015 at Reina Sofía Hospital (Córdoba, Spain). The primary objective of the CTOXALL study was to investigate the long-term prevalence of cardiotoxicity in CLS. Previously, we have reported a high prevalence of left ventricular systolic and diastolic abnormalities in this cohort of patients [6,12]. The current study is focused on the assessment of the RV, adding an automated RVS quantification to standard echocardiographic parameters.

The research protocol for the CTOXALL study received approval from the local clinical research ethics committee in accordance with institutional and good clinical practice guidelines. Written informed consent was obtained from all participants, as well as from the parents or legal guardians of minors involved.

Participants underwent evaluation between May 2019 and January 2022. Eligible participants were survivors diagnosed with ALL before the age of 18 and had received the last anthracycline dose at least 3 years before their inclusion in this study. An individual with a congenital heart disease was excluded. A comparison group consisting of healthy siblings of the survivors who were willing to participate was also recruited.

### 2.2. Clinical Assessment

A comprehensive clinical assessment was performed on all patients. Information related to cardiotoxic treatment exposure, including chemotherapeutic agents and dosage, radiotherapy exposure, and hematopoietic stem-cell transplantation (HSCT), was retrieved from medical records. Cumulative anthracycline dose was expressed as doxorubicin equivalents (mg/m^2^), which were computed using previously described conversion factors: 0.6× daunorubicin dose, 0.8× epirubicin dose, and 10.5× mitoxantrone [13]. Radiotherapy exposure was considered when it encompassed the heart region, including total body irradiation.

### 2.3. Echocardiography

Data from conventional echocardiographic parameters were available from the original CTOXALL study. For the present study, a post hoc measurement of RVS was conducted. All examinations were conducted by certified cardiologists using either an EPIQ CVx or iE33 echocardiographic system (Philips Medical Systems, Andover, MA, USA). Standard echocardiographic parameters were acquired in adherence to the most recent guidelines [14,15]. The echocardiographic study included the following right ventricular systolic function measurements: tricuspid annular plane systolic excursion (TAPSE), fractional area change (FAC), doppler tissue imaging tricuspid annular systolic peak-wave velocity (s’DTI), right ventricular free-wall strain (RVFWS), and right ventricular four-chamber strain (RV4CS). Strain parameters were quantified from an RV-focused apical four-chamber view using a semi-automated assessment with AutoStrain (TomTec-Arena, TomTec Imaging Systems, Munich, Germany). Manual adjustment of the software’s automatic endocardial border delineation was permitted. The cardiologists responsible for analyzing the strain parameters were unaware of each participant’s group.

**Figure 1:**
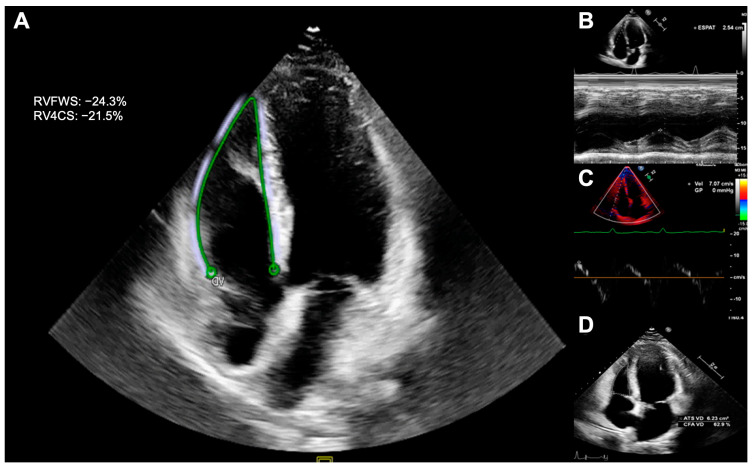
Echocardiographic assessment of right ventricular systolic function. Right ventricular strain (**A**), tricuspid annular plane systolic excursion (**B**), s’DTI (**C**), and fractional area change (**D**).

The cut-off values for right ventricular systolic dysfunction (RVSD) were as follows: TAPSE < 17 mm, FAC < 35%. s’DTI < 9.5 cm/s, and RVFWS < 20% (Figure 1) [15,16]. For simplicity, RVS measurements are reported as their absolute values.

### 2.4. Variability Analysis

To evaluate the intraobserver and interobserver variability in RVFWS, TAPSE, and FAC measurements, the analysis of 20 randomly selected studies was repeated by the same investigator who performed the analysis and another investigator.

### 2.5. Statistical Analysis

Descriptive data are presented as the count (percentage) for categorical variables and, according to the distribution, as the mean ± standard deviation or median and interquartile range (25th–75th percentiles) for continuous variables. Normal distribution was assessed via the Shapiro–Wilk test and QQ plots. Between-group comparisons of descriptive data were performed via the chi-square test or the Fisher exact test for categorical variables and the Student *t*-test or the Mann–Whitney U test for continuous variables, as appropriate.

Between-group comparisons of echocardiographic measurements were performed using linear regression models. The inverse probability of treatment weighting (IPW) was used to address confounding [17]. Propensity scores were calculated with a logistic regression model that included the group as the dependent variable and age, sex, body mass index, heart rate, and diastolic blood pressure as covariates. To assess covariate balance, standardized mean differences were computed both before and after applying the weighting. A difference of less than 0.1 was considered indicative of good balance. Standard errors of the IPW linear regression coefficients were obtained using robust sandwich-type variance estimators [18].

Multivariable linear regression analyses were conducted to determine significant predictors for TAPSE, FAC, and RVFWS in the CLS. Covariates included sex, age, age at diagnosis, time since diagnosis, heart rate, systolic and diastolic blood pressure, hypertension, hypercholesterolemia, diabetes mellitus, obesity, smoking, sedentarism, cumulative anthracycline dose, radiotherapy, and HSCT. The models were tested for collinearity and were built using backward stepwise elimination, initially including clinically relevant variables and those with a *p* < 0.100 in the univariable models.

Intraobserver and interobserver variability were assessed through intraclass correlation coefficients and the Bland–Altman method, plotting the differences of 2 measurements (*y*-axis) against their mean (*y*-axis) for each subject. The limit of agreement was computed as the mean difference ± 1.96 standard deviations [19].

Statistical analyses were performed using the SPSS software (version 24; IBM Corp., Armonk, NY, USA) and R software (version 4.2.1; R Foundation for Statistical Computing, Vienna, Austria).

## 3. Results

### 3.1. Baseline Characteristics

For the present study, all 148 participants from the original CTOXLAL study were analyzed: 90 CLS and a control group comprising 58 healthy siblings. The CLS had a mean age at inclusion of 24.6 ± 9.7, and 34 (37.8%) were female. Table 1 displays the clinical characteristics of the groups. Both groups were similar in terms of age, body measurements, and the prevalence of risk factors. However, the control group had a higher proportion of female participants (56.6%; *p* = 0.018). Sedentarism was prevalent in both groups (41.1% vs. 34.5%; *p* = 0.525), as was obesity (10.0% vs. 12.1%, *p* = 0.901). Smoking was more common in the CLS group (15.6% vs. 1.7%; *p* = 0.005).

### 3.2. Cardiotoxic Treatment Exposure

The median age at diagnosis was 4 (3–8) years, and the median time elapsed from diagnosis to recruitment was 18 (11–26) years. All the survivors were exposed to anthracyclines, but only three (3.3%) received radiotherapy. Treatment details are shown in Table 1. According to the treatment risk categories for childhood and adolescent cancer survivors proposed in the European Society of Cardiology Cardio-Oncology guidelines [20], 23 (25.6%), 61 (67.7%), 3 (3.3%), and 3 (3.3%) CLS could be classified as low, moderate, high risk, and very high risk, respectively.

### 3.3. Prevalence of Right Ventricular Systolic Dysfunction

**Figure 2:**
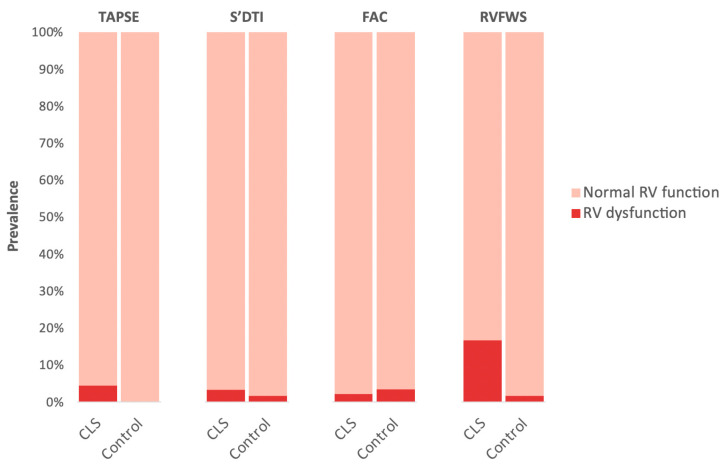
Prevalence of right ventricular systolic dysfunction. Cut-off points: TAPSE < 17 mm, FAC < 35%, s’DTI < 9.5 cm/s, and RVFWS < 20%. FAC: fractional area change. RVFWS: right ventricular free-wall strain; TAPSE: tricuspid annular plane systolic excursion.

The prevalence of right ventricular systolic dysfunction in CLS was relatively low, ranging from 2.2 to 4.4%, and did not significantly differ from the rates observed in the control group when considering conventional right ventricular systolic parameters (TAPSE, FAC, S’DTI). However, the prevalence of subclinical right ventricular systolic dysfunction, as determined using the RVFWS, was significantly higher in the CLS cohort (16.7% vs. 1.7%, *p* = 0.005), especially in those with left ventricular systolic dysfunction (*n* = 11) (36.4% vs. 13.9%, *p* = 0.082) or subclinical left ventricular systolic dysfunction (*n* = 24) (37.5% vs. 9.1%, *p* = 0.001). Detailed prevalence rates for each parameter are presented in Figure 2.

### 3.4. Comparison of Echocardiographic Parameters between the Groups

**Figure 3:**
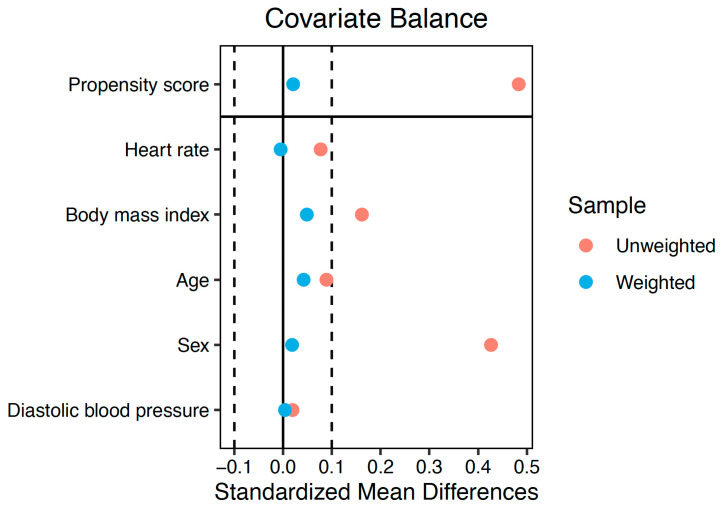
Covariate balance of the covariables included in the propensity score model. In the weighted pseudo-population (blue), all the variables showed an excellent balance with standardized mean differences <0.1.

On average, the CLS group exhibited lower values for the majority of right ventricular systolic function measurements, including TAPSE, RVFWS, and RV4CS. Conversely, FAC and S’DTI were comparable to the control group. The peak systolic RV-RA gradient was slightly higher in the CLS group. Of note, average right ventricular systolic function parameters were within the normal range in both groups. Right ventricular systolic function echocardiographic parameters, along with additional echocardiographic measurements, are summarized in Table 2. In addition to the unadjusted comparisons, the estimated differences in the IPW population are shown in the table. The covariables included in the propensity score model were well balanced after the weighting, with standardized mean differences <0.1 for all of them (Figure 3). In the IPW-adjusted comparisons, TAPSE, RVFWS, and RV4CS were also decreased compared to the control group. Moreover, CLS RV had lower values of RV end-diastolic area (beta: −1.43 cm^2^, *p* = 0.042).

### 3.5. Predictors of Conventional Right Ventricular Systolic Function Measurements in the Survivors

The results of univariable and multivariable linear regression models for TAPSE are presented in Table 3. In the univariable analysis, female sex, age at examination, time since diagnosis, heart rate, and HSCT history were associated with TAPSE. In the multivariable model, the only factors that were associated with TAPSE were HSCT history (Beta: −2.72, 95% CI: 4.70 to −0.65, *p* = 0.01) and age at examination (Beta: 0.10, 95% CI: 0.02 to 0.18, *p* = 0.01).

Univariable and multivariable linear regression models for FAC are shown in Table 4. In the univariable analysis, diabetes mellitus was the only factor associated with FAC (Beta: −9.71, 95% CI: −19.04 to −0.42, *p* = 0.41). In the multivariable model, which included other clinically relevant variables, this association was not significant (*p* = 0.74).

### 3.6. Predictors of Right Ventricular Free-Wall Strain in the Survivors

Univariable and multivariable linear regression models for RVFWS are presented in Table 5. In the univariable analysis, obesity, sedentarism, and smoking habits were associated with RVFWS. In the multivariable model, obesity (Beta: −5.12, 95% CI: −9.41 to −0.76, *p* = 0.02) and smoking (Beta: −3.17, 95% CI: −5.80 to −0.34, *p* = 0.02) were independently associated with RVFWS.

### 3.7. Intraobserver and Interobserver Variability Analysis

**Figure 4:**
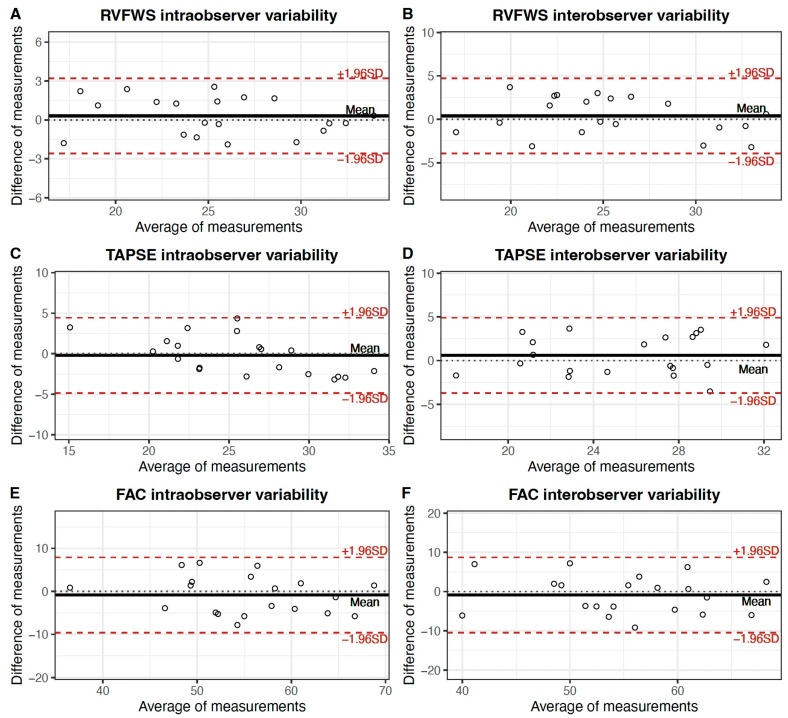
Bland–Altman plots: RVFWS intraobserver variability (**A**); RVFWS interobserver agreement (**B**); TAPSE intraobserver agreement (**C**); TAPSE interobserver agreement (**D**); FAC intraobserver agreement (**E**); FAC interobserver agreement (**F**). FAC: fractional area change. RVFWS: right ventricular free-wall strain; TAPSE: tricuspid annular plane systolic excursion.

The variability analysis revealed a high level of agreement in the measured RV systolic function parameters, with particular robustness observed in RVFWS. The intraclass correlation coefficients for intraobserver agreement were 0.95 (95% CI: 0.89 to 0.98) for RVFWS, 0.89 (95% CI: 0.74 to 0.96) for TAPSE, and 0.85 (95% CI: 0.66 to 0.94) for FAC. For interobserver agreement, the intraclass correlation coefficients values were 0.90 (95% CI: 0.77 to 0.96) for RVFWS, 0.85 (95% CI: 0.67 to 0.94) for TAPSE, and 0.81 (95% CI: 0.58 to 0.92) for FAC. Bland–Altman plots (Figure 4) were consistent with these findings, showing acceptable agreement intervals and no relevant bias.

## 4. Discussion

In this study, we evaluated RV function in a cohort of long-term survivors of childhood ALL using conventional echocardiographic measurements and RVS. The main findings were as follows: (1) the prevalence of RVSD was notably higher when assessed with RVS compared to conventional parameters; (2) most RV systolic parameters showed significant reductions compared to the control group; (3) modifiable cardiovascular risk factors were frequent and inversely associated with RVS.

### 4.1. Prevalence of Right Ventricle Dysfunction

While historically overlooked, there has recently been a renewed interest in the study of the RV and its significance in cardiovascular disease [21]. In the context of cardio-oncology, although far less explored than the left ventricle, there is evidence supporting the susceptibility of the RV to anthracycline-induced acute cardiotoxicity [22]. However, scarce data are available regarding RV involvement in long-term survivors of childhood cancer with prior anthracycline exposure.

In the present study, we found a relatively low prevalence of RVSD using conventional 2D echocardiographic parameters, ranging from 2.2 to 4.4%. Nevertheless, when RV function was assessed with RVFWS, the prevalence of RVSD increased to 16.7%. In line with our findings, previous studies have reported a prevalence of RVSD that varied between 6 and 28%, depending on the diagnostic tool, the cut-off values, and the characteristics of the study sample, especially those related to treatment exposure (age, anthracycline dose, and proportion of patients exposed to radiotherapy). Massey et al. found a prevalence of RVSD of 13.6% in a cohort of survivors of HSCT treated in their youth (17.6 ± 9.5 years at HSCT) [23]. The prevalence rate is in line with our findings, and the findings of this study support the observed association between HSCT and TAPSE reduction in the multivariable model. Murbraech et al. reported a prevalence of RVSD of 6.2% in a cohort of adult long-term lymphoma survivors, with a mean age at diagnosis of 42 years [24]. Despite the higher cumulative anthracycline dose and more frequent exposure to radiotherapy, the lower prevalence rate observed in their study could be attributed to the application of stricter criteria for RVSD (requiring impairment in at least two parameters) or, potentially, to the different age profiles of the cohorts, since myocardia have been proposed to be especially vulnerable to anthracycline-induced injury during childhood. On the other hand, Christiansen et al. found a prevalence of RVSD of 28.2% [8]. The underlying reason for this noticeable high prevalence may lie in the more intensive treatment received by the recruited survivors: the cumulative anthracycline dose was 150 (40–485) mg/m^2^, and 24% of them were exposed to radiotherapy, which appears to exert a particularly cardiotoxic effect on the RV due to its anterior anatomical position [25]. In addition, the lower limits of normal RV function were based on the distribution of the control group values, resulting in more lenient criteria for RV impairment. Consistent with these results, Ylanen et al. reported a RVSD prevalence of 27% in a cohort of childhood cancer survivors using cardiac magnetic resonance (CMR), the gold standard for RV assessment [26]. The median cumulative anthracycline dose was remarkably high, i.e., 222 mg/m^2^ (80–419 mg/m^2^), and 11% received radiotherapy. Interestingly, no myocardial fibrosis, as assessed via late gadolinium enhancement, was detected. Although CMR was not available in our study, RVFWS has been previously demonstrated to be strongly correlated with CMR-based RV function [11,27,28].

### 4.2. Comparison of RV Echocardiographic Parameters between Survivors and Healthy Siblings

The CLS group presented lower values for the majority of right ventricular systolic function parameters, including TAPSE, RVFWS, and RV4CS, even after adjusting for potential confounders by means of IPW models. These findings are similar to those reported in other previously detailed cohorts of long-term survivors that included a control group [8,24]. Consistent with our results, the reported average values, although reduced compared to controls, were within the normal range. These data support the causal effect of anthracyclines on RV function in long-term survivors of childhood cancers, who might present subtle alterations in RV systolic function that may not reach the threshold for a RVSD diagnosis. In this context, RVFWS, capable of detecting RVSD at earlier stages, appears to be of paramount importance [16].

### 4.3. Association of RV Subclinical Dysfunction with Cardiovascular Risk Factors

In long-term childhood cancer survivors, the presence of modifiable cardiovascular risk factors is frequent and can significantly potentiate the risk of cardiovascular disease associated with cardiotoxic therapies [29]. The reason behind the increased frequency of cardiovascular risk factors is not clearly established, but it appears to be related to psychological factors such as stress and emotional distress [30]. Among participants in the CTOXALL cohort, a considerable prevalence of obesity (10%), sedentary lifestyle (41.1%), and smoking habits (15.6%) was observed. Furthermore, all three variables were associated with RVFWS in the univariable analysis, and obesity and smoking were independently associated with RVFWS in the multivariable model. These findings align with the recommendations outlined in the 2022 European Society of Cardiology Guidelines on cardio-oncology, emphasizing the importance of promoting healthy lifestyle habits and advocating for the annual screening of modifiable cardiovascular risk factors in these patients [20]. Such preventive measures take on particular significance given the observed association between these factors and RVFWS.

### 4.4. Limitations

The current study has several limitations. Firstly, RV was not evaluated with CMR to complement our findings with tissue characterization. Secondly, the cross-sectional design prevents us from identifying the exact moment of the RVSD onset. Thirdly, the conformation of the control group with healthy siblings resulted in sex and smoking habit differences between the two groups. Smoking was not included in the PS model because it was only present in one participant in the control group. However, sex was well balanced in the weighted population, while the inclusion of siblings as controls may have helped to reduce unmeasured confounding related to demographic, sociocultural, environmental, and genetic factors. Lastly, echocardiographic measurements were not analyzed by a core laboratory team.

## 5. Conclusions

In this cohort of long-term ALL survivors, right ventricular subclinical dysfunction was present in one out of every six patients. When compared to healthy siblings, most RV systolic parameters showed reductions. Notably, obesity and smoking were associated with greater reductions in RVFWS in the survivors.

## Figures and Tables

**Table 1:** Characteristics of childhood leukemia survivors and controls.

	CLSs (*n* = 90)	Control Group (*n* = 58)	*p*-Value
**Clinical characteristics**			
Age at diagnosis (years)	4 (3–7)	-	-
Age at exam (years)	24.6 ± 9.7	23.6 ± 10.8	0.593
Time since diagnosis (years)	18 (11–26)	-	-
Sex (% female)	34 (37.8%)	34 (58.6%)	0.018
Weight (kg)	64.8 ± 18.3	61.9 ± 17.2	0.333
Height (cm)	165.6 ± 13.3	164.2 ± 13.8	0.539
Body mass index (kg/m^2^)	23.3 ± 5.1	22.6 ± 4.4	0.346
Body surface area (m^2^)	1.7 ± 0.3	1.7 ± 0.3	0.366
Systolic blood pressure (mmHg)	116.2 ± 11.3	115.6 ± 11.1	0.768
Diastolic blood pressure (mmHg)	69.6 ± 7.9	69.4 ± 7.9	0.908
Heart rate (bpm)	72.5 ± 11.1	71.7 ± 11.5	0.646
Current smoker (%)	14 (15.6%)	1 (1.7%)	0.005
Hypertension (%)	3 (3.3%)	0 (0.0%)	0.280
Hypercholesterolemia (%)	12 (13.3%)	4 (6.9%)	0.283
Diabetes mellitus (%)	4 (4.4%)	1 (1.7%)	0.649
Obesity	9 (10.0%)	7 (12.1%)	0.901
Sedentarism (%)	37 (41.1%)	20 (34.5%)	0.525
**Cardiotoxic therapies**			
Anthracycline dose (mg/m^2^)	138 (72–192)	-	-
Radiotherapy (%)	3 (3.3%)	-	-
**ESC guidelines risk category**			
Low risk	23 (25.6%)		
Moderate risk	61 (67.7%)		
High risk	3 (3.3%)		
Very high risk	3 (3.3%)		

ALL: acute lymphoblastic leukemia; CLSs: childhood acute lymphoblastic leukemia survivors. ESC: The European Society of Cardiology. HSCT: hematopoietic stem cell transplantation.

**Table 2:** Echocardiographic parameters of childhood leukemia survivors and controls.

	CLSs (*n* = 90)	Control Group (*n* = 58)	*p*-Value	IPW Beta (RSE)	P_IPW_-Adjusted
**Right ventricle**					
End-diastolic area (cm^2^)	16.5 ± 4.0	17.4 ± 3.9	0.185	−1.43 (0.69)	0.042
End-systolic area (cm^2^)	8.1 ± 3.1	8.7 ± 2.9	0.277	−0.94 (0.52)	0.071
Fractional area change (%)	52.0 ± 9.3	51.2 ± 6.9	0.570	1.45 (1.38)	0.293
TAPSE (mm)	23.3 ± 4.0	25.2 ± 3.4	0.004	2.08 (0.64)	0.001
S’ DTI (cm/s)	13.8 ± 2.4	14.4 ± 2.6	0.196	0.73 (0.49)	0.136
RVFWS (-%)	24.9 ± 4.6	26.8 ± 4.7	0.032	1.92 (0.85)	0.025
RV4CS (-%)	21.7 ± 3.3	23.1 ± 3.4	0.017	1.57 (0.64)	0.016
RV-RA gradient (mmHg)	19.2 ± 4.9	16.1 ± 3.9	0.024	3.15 (1.39)	0.027
**Left ventricle**					
LVDD (mm)	45.6 ± 6.8	44.9 ± 6.1	0.595	0.09 (1.14)	0.935
LVSD (mm)	28.9 ± 6.0	26.2 ± 4.6	0.005	2.27 (0.92)	0.015
IVS (mm)	7.6 ± 1.5	7.7 ± 1.3	0.824	0.17 (0.23)	0.457
LVEF 2D (%)	56.2 ± 5.8	62.4 ± 5.5	<0.001	5.45 (0.95)	<0.001
GLS (-%)	20.4 ± 2.8	22.9 ± 2.3	<0.001	2.28 (0.45)	<0.001

CLSs: childhood acute lymphoblastic leukemia survivors; IPW: inverse probability weighting; RSE: robust standard error; Gradient RV-RA: pressure gradient between the right ventricle and the right atrium; TAPSE: tricuspid annular plane systolic excursion; S’ DTI: tissue Doppler-derived right ventricular systolic excursion velocity S’. RVFWS: right ventricular free-wall strain; RV4CS: right ventricular four-chamber strain; LVDD: left ventricular diastolic diameter; LVSD: left ventricular systolic diameter; IVS: interventricular septum; LVEF: left ventricular ejection fraction; GLS: global longitudinal strain.

**Table 3:** Univariable and multivariable regression models for TAPSE in the survivors.

	Univariable			Multivariable		
	Beta	95% CI	*p*-Value	Beta	95% CI	*p*-Value
Sex (female)	1.76	0.05 to 3.48	0.044	1.41	−0.28 to 3.03	0.102
Age at diagnosis (years)	0.02	−0.16 to 0.21	0.815			
Age at exam (years)	0.09	0.00 to 0.17	0.042	0.10	0.02 to 0.18	0.017
Time since diagnosis (years)	0.10	0.01 to 0.19	0.032			
HR (bpm)	−0.08	−0.157 to −0.01	0.029			
SBP (mmHg)	−0.02	−0.09 to 0.06	0.634			
DBP (mmHg)	0.01	−0.09 to 0.12	0.808			
Hypertension	3.36	−1.27 to 7.99	0.153			
Hypercholesterolemia	0.65	−1.82 to 3.13	0.602			
Diabetes mellitus	−0.73	−4.81 to 3.35	0.723			
Obesity	1.04	−1.76 to 3.84	0.462			
Sedentarism	−0.08	−1.80 to 1.65	0.928			
Current smoker	−0.93	−3.25 to 1.38	0.425			
Anthracycline dose	−0.01	−0.02 to 0.01	0.239			
Radiotherapy	−4.02	−8.63 to 0.58	0.086			
HSCT	−2.42	−4.52 to −0.33	0.024	−2.72	−4.70 to −0.65	0.010

CI: confidence interval, HR: heart rate, SBP: systolic blood pressure, DBP: diastolic blood pressure, HSCT: hematopoietic stem-cell transplantation.

**Table 4:** Univariable and multivariable regression models for FAC in the survivors.

	Univariable			Multivariable		
	Beta	95% CI	*p*-Value	Beta	95% CI	*p*-Value
Sex (female)	−0.43	−4.47 to 3.62	0.835	−0.38	−4.66 to 3.89	0.856
Age at diagnosis (years)	0.05	−0.38 to 0.483	0.811			
Age at exam (years)	−0.02	−0.23 to 0.178	0.818	0.01	−0.22 to 0.23	0.961
Time since diagnosis (years)	−0.05	−0.27 to 0.180	0.690			
HR (bpm)	0.04	−0.14 to 0.219	0.645			
SBP (mmHg)	0.10	−0.07 to 0.273	0.254			
DBP (mmHg)	−0.05	−0.29 to 0.197	0.687			
Hypertension	−8.70	−19.51 to 2.08	0.112			
Hypercholesterolemia	−0.86	−6.63 to 4.91	0.768			
Diabetes mellitus	−9.71	−19.04 to −0.42	0.041	−9.34	−20.35 to 0.93	0.074
Obesity	−3.70	−10.2 to 2.79	0.261	−2.57	−9.51 to 4.49	0.470
Sedentarism	0.04	−3.95 to 4.03	0.984			
Current smoker	3.51	−1.85 to 8.87	0.197	3.28	−2.54 to 8.92	0.264
Anthracycline dose	0.01	−0.02 to 0.04	0.378	0.02	−0.01 to 0.05	0.248
Radiotherapy	−3.78	−14.72 to 7.12	0.493	−2.12	−14.12 to 9.43	0.723
HSCT	−2.79	−7.77 to 2.18	0.267			

CI: confidence interval, HR: heart rate, SBP: systolic blood pressure, DBP: diastolic blood pressure, HSCT: hematopoietic stem-cell transplantation.

**Table 5:** Univariable and multivariable regression models for RVFWS in the survivors.

	Univariable			Multivariable		
	Beta	95% CI	*p*-Value	Beta	95% CI	*p*-Value
Sex (female)	−0.13	−2.23 to 1.97	0.900	0.40	−1.70 to 2.50	0.703
Age at diagnosis (years)	−0.05	−0.29 to 0.20	0.710			
Age at exam (years)	−0.07	−0.17 to 0.04	0.223	0.01	−0.10 to 0.12	0.831
Time since diagnosis (years)	−0.07	−0.19 to 0.05	0.257			
HR (bpm)	−0.04	−0.13 to 0.05	0.363			
SBP (mmHg)	0.00	−0.09 to 0.09	0.940			
DBP (mmHg)	0.09	−0.05 to 0.22	0.196			
Hypertension	−1.18	−7.76 to 5.40	0.722			
Hypercholesterolemia	−1.16	−4.13 to 1.82	0.441			
Diabetes mellitus	1.39	−5.19 to 7.96	0.676			
Obesity	−5.09	−9.18 to −1.00	0.015	−5.12	−9.41 to −0.76	0.022
Sedentarism	−2.46	−4.46 to −0.45	0.017			
Current smoker	−3.25	−5.85 to −0.64	0.015	−3.17	−5.80 to −0.34	0.028
Anthracycline dose	−0.01	−0.02 to 0.01	0.527	−0.01	−0.02 to 0.01	0.312
Radiotherapy	−2.70	−8.07 to 2.68	0.321	−3.01	−8.32 to 2.33	0.264
HSCT	−1.73	−4.26 to 0.81	0.179			

CI: confidence interval, HR: heart rate, SBP: systolic blood pressure, DBP: diastolic blood pressure, HSCT: hematopoietic stem-cell transplantation.

## Data Availability

The data presented in this study are available on request from the corresponding author.
